# Modification of Cassava Root Starch Phosphorylation Enhances Starch Functional Properties

**DOI:** 10.3389/fpls.2018.01562

**Published:** 2018-10-30

**Authors:** Wuyan Wang, Carmen E. Hostettler, Fred F. Damberger, Jens Kossmann, James R. Lloyd, Samuel C. Zeeman

**Affiliations:** ^1^Institute of Molecular Plant Biology, Department of Biology, ETH Zürich, Zürich, Switzerland; ^2^Institute of Molecular Biology and Biophysics, Department of Biology, ETH Zürich, Zürich, Switzerland; ^3^Biomolecular NMR Spectroscopy Platform, Department of Biology, ETH Zürich, Zürich, Switzerland; ^4^Department of Genetics, Institute for Plant Biotechnology, Stellenbosch University, Stellenbosch, South Africa

**Keywords:** *Manihot esculenta* Crantz, glucan water dikinase (GWD), starch excess 4 (SEX4), like sex four 2 (LSF2), gene overexpression, RNAi silencing

## Abstract

Cassava (*Manihot esculenta* Crantz) is a root crop used as a foodstuff and as a starch source in industry. Starch functional properties are influenced by many structural features including the relative amounts of the two glucan polymers amylopectin and amylose, the branched structure of amylopectin, starch granule size and the presence of covalent modifications. Starch phosphorylation, where phosphates are linked either to the C3 or C6 carbon atoms of amylopectin glucosyl residues, is a naturally occurring modification known to be important for starch remobilization. The degree of phosphorylation has been altered in several crops using biotechnological approaches to change expression of the starch-phosphorylating enzyme GLUCAN WATER DIKINASE (GWD). Interestingly, this frequently alters other structural features of starch beside its phosphate content. Here, we aimed to alter starch phosphorylation in cassava storage roots either by manipulating the expression of the starch phosphorylating or dephosphorylating enzymes. Therefore, we generated transgenic plants in which either the wild-type potato GWD (*StGWD*) or a redox-insensitive version of it were overexpressed. Further plants were created in which we used RNAi to silence each of the endogenous phosphoglucan phosphatase genes STARCH EXCESS 4 (*MeSEX4*) and LIKE SEX4 2 (*MeLSF*), previously discovered by analyzing leaf starch metabolism in the model species *Arabidopsis thaliana*. Overexpressing the potato GWD gene (*StGWD*), which specifically phosphorylates the C6 position, increased the total starch-bound phosphate content at both the C6 and the C3 positions. Silencing endogenous LSF2 gene (*MeLSF2*), which specifically dephosphorylates the C3 position, increased the ratio of C3:C6 phosphorylation, showing that its function is conserved in storage tissues. In both cases, other structural features of starch (amylopectin structure, amylose content and starch granule size) were unaltered. This allowed us to directly relate the physicochemical properties of the starch to its phosphate content or phosphorylation pattern. Starch swelling power and paste clarity were specifically influenced by total phosphate content. However, phosphate position did not significantly influence starch functional properties. In conclusion, biotechnological manipulation of starch phosphorylation can specifically alter certain cassava storage root starch properties, potentially increasing its value in food and non-food industries.

## Introduction

Starch is one of the most important plant products. It is the major nutritive component of our major staple crops, including cereals such as maize, wheat and rice, as well as root crops like potato and cassava. In addition to its nutritional value, starch has a myriad of uses in the food and non-food industries (e.g., as a texturizer, an adhesive, a coating agent, a floculant, a component of biodegradable plastics, a feedstock for sugar and ethanol production). In order to fulfill these different functional uses, starch and starch derivatives need to have distinct physicochemical properties (e.g., solubility, viscosity, film-forming ability). Functional diversity is obtained partly by using starches from different botanical sources and partly through chemical, physical and enzymatic treatments, performed on starch after its extraction/gelatinisation to modify the constituent polymers (Singh et al., 2003; Santelia and Zeeman, 2011; Alcázar-Alay and Meireles, 2015).

Cassava (*Manihot esculenta* Crantz) is one of the worlds major starch crops. It is a perennial shrub in the Euphorbiaceae family and is commercially cultivated in tropical and subtropical regions (Allem and Genéticos, 2002; Alves, 2002; Puounti-Kaerlas, 2002; El-Sharkawy, 2004). Its swollen storage roots are rich in starch, and represent an important food source for hundreds of millions of people. In South and Southeast Asia, 40% of the cassava harvest is used to produce extracted starch and in 2014, the international trade of cassava starch and flour was estimated at 8.5 million tons (Karlström et al., 2016). In the food industry, cassava starch offers certain advantages over other starches as a texturizer. First, it is relatively inexpensive. Second, it has a low gelatinization temperature and produces relatively clear, high-viscosity pastes. Third, its bland taste makes it preferable as additive for processed food with mild flavors (Raphael et al., 2011; Vasconcelos et al., 2016). In non-food industries, cassava starch is used in fuel-ethanol production, in paper and textile production, and in the pharmaceutical industry as an inert carrier (Balagopalan, 2002; Breuninger et al., 2009).

Starch is composed primarily of two glucose polymers – amylose and amylopectin. Amylopectin is the major polymer constituting 70% or more of wild type starches. The glucosyl units of starch are α-1,4-linked to form chains that are connected by α-1,6-bonds, yielding a tree-like or racemose structure. The branches of amylopectin are clustered and interact to form double helices that pack into semi-crystalline lamellae, resulting in highly ordered, insoluble starch granules (Zeeman et al., 2010; Pfister and Zeeman, 2016). Amylose is the minor component constituting the remaining 30% or less of starch. Both the structure of amylopectin and the relative amounts of amylopectin and amylose are major determinants of the functional properties of starch (Alcázar-Alay and Meireles, 2015).

Glucan phosphorylation is an important, naturally occurring starch modification which is known to increase the hydration capacity and properties of starch pastes (Jobling, 2004; Carpenter et al., 2015). In wild-type starches, phosphate groups are bound primarily to the C6 position of amylopectin glucosyl residues, with smaller amounts bound to the C3 position. In the past two decades, the enzymes responsible for the reversible phosphorylation of starch in plants were discovered and this process was shown to play an important role in starch metabolism. Phosphorylation is mediated by two dikinases, namely Glucan Water Dikinase (GWD) and Phosphoglucan Water Dikinase (PWD) (Lorberth et al., 1998; Yu et al., 2001; Baunsgaard et al., 2005; Kötting et al., 2005). GWD and PWD both transfer the β-phosphate group of ATP to amylopectin glucosyl residues, phosphorylating the C6 and C3 positions, respectively. The γ-phosphate group of ATP is released as orthophosphate (Ritte et al., 2002, 2006). For PWD to function, it requires amylopectin to have been previously phosphorylated by GWD (Baunsgaard et al., 2005; Kötting et al., 2005). Thus, starch from *gwd* knockout mutants are phosphate free, whereas *pwd* mutant starches lack phosphate at the C3 position. Two phosphoglucan phosphatases responsible for amylopectin dephosphorylation have also been identified, namely Starch EXcess 4 (SEX4) and Like-SEX Four 2 (LSF2), mainly through work on transitory leaf starch in Arabidopsis. SEX4 releases phosphate from both the C6 and C3 positions of amylopectin, with the preference for C6. In contrast, LSF2 is specific for C3-bound phosphate (Gentry et al., 2007; Kötting et al., 2009; Santelia et al., 2011; Silver et al., 2014).

Although some starch phosphorylation proceeds concurrently with starch synthesis, the biological role of phosphorylation is thought to be the initiation of starch degradation in living cells. The lack of GWD or PWD results in *starch-excess* phenotypes in leaves and retardation in vegetative and/or reproductive growth, which is particularly severe in the case of *gwd* mutants (Caspar et al., 1991; Lorberth et al., 1998; Yu et al., 2001; Nashilevitz et al., 2009; Weise et al., 2012; Hirose et al., 2013). Phosphorylation is proposed to disrupt the semi-crystalline packing of amylopectin, making the glucan chains at the granule surface more accessible to glucan degrading enzymes such as amylases (Edner et al., 2007; Hejazi et al., 2009). Phosphoglucan phosphatases are also required for normal rates of starch degradation. The loss of SEX4 also causes a *starch-excess* phenotype in leaves and decreases plant growth (Zeeman et al., 1998; Kötting et al., 2009), which is exacerbated by the additional loss of LSF2. This is explained by the fact that phosphate groups interfere with the full degradation of starch by blocking the action of β-amylases. Thus, after disrupting the semi-crystalline parts of starch, the phosphate groups need to be removed again prior to complete amylolysis (Kötting et al., 2009; Hejazi et al., 2010). Consequently, phosphoglucan phosphatase-deficient mutants accumulate phosphorylated intermediates of starch degradation, or have increased levels of starch-bound phosphate (Kötting et al., 2009; Santelia et al., 2011). Both GWD and SEX4 have been reported to be redox-regulated enzymes, based on the formation of an intramolecular disulfide bond between two cysteines (Mikkelsen et al., 2005; Silver et al., 2013). When reduced, the enzymes are active whereas they are inactive in the oxidized state. In the case of potato GWD, substitution of one of the cysteines with a serine resulted in an active, redox-insensitive enzyme (Mikkelsen et al., 2005).

Various phosphate levels have been reported for starches from different species and different plant organs (Blennow et al., 1998). Storage starch from cereal seeds has very low phosphate (less than 1 nMol Glc-6-P/mg starch, equivalent to less than 1 phosphate per 6000 glucose residues) compared to other starches. In contrast, potato tuber starch has particularly high levels of starch-bound phosphate (8–33 nMol Glc-6-P/mg starch, equivalent to more than 1 phosphate per 200 glucose residues), while the level in cassava root starch is rather low (2.5 nMol Glc-6-P/mg starch). The discovery of the starch phosphorylating/dephosphorylating enzymes has opened the door to engineering starch phosphate levels and starch content in crops through manipulating their expression. This has been applied in potato (*Solanum tuberosum*), barley (*Hordeum vulgare*), maize (*Zea mays*), wheat (*Triticum aestivum*), and rice (*Oryza sativa*) (Lorberth et al., 1998; Schewe et al., 2003; Lanahan and Basu, 2005; Carciofi et al., 2011; Chen et al., 2017; Xu et al., 2017a,b) and was particularly effective in species with low starch-bound phosphate. For example, an almost tenfold increase in grain starch-bound phosphate was achieved by heterologous overexpression of *StGWD* in barley endosperm. This high phosphate level was accompanied by altered starch granule morphology, composition and thermal properties, enhancing economically important traits (Carciofi et al., 2011; Chen et al., 2017; Xu et al., 2017a). Such biotechnological approaches are potentially valuable in both agriculture and industry, potentially increasing the value of starch and decreasing the need for post-extraction modifications.

To alter starch phosphorylation in the storage root starch of cassava, transgenic plants were generated in which we either overexpressed the potato GWD gene (*StGWD*; both the wild-type protein and a redox-insensitive version of it), or silenced each of the endogenous phosphoglucan phosphatase genes (*MeSEX4* and *MeLSF2*). Both approaches altered starch-phosphorylation in the predicted way resulting in starches with distinct properties.

## Materials and Methods

### Generation of Transgenic Lines

Cassava (*Manihot esculenta* cv 60444) was used as the wild type. For overexpression of GWD, two constructs harboring coding sequence of either the wild-type potato GWD (pCAMBIA2300::*StGWD*) or a redox-insensitive version of it (pCAMBIA2300::*StGWD*_m_) were obtained from Mikkel Glaring (University of Copenhagen, Copenhagen, Denmark). The redox-insensitive version contains a nucleotide modification leading to an amino acid substitution of cysteine 1084 to a serine (C1084S) at the peptide level. For RNA interference lines (RNAi), hairpin constructs targeting either the *MeSEX4* or the *MeLSF2* genes were designed. The protein sequences of *At*SEX4 and *At*LSF2 were used as a query to identify orthologous proteins in cassava (Manes.10G053500.1 and Manes.10G005000.1, respectively ^1^). Hairpin constructs containing an antisense fragment, a loop, and a sense fragment designed for *MeSEX4* or *MeLSF2*, were subcloned into a modified pCAMBIA1301 vector containing the *S. tuberosum* patatin B33 promoter. Transgenic cassava lines were obtained via Agrobacterium-mediated friable embryonic callus (FEC) transformation as described in (Bull et al., 2009; Niklaus et al., 2011). Transgenic plantlets selected by antibiotic resistance and confirmed for the presence of the transgene by PCR on genomic DNA were transferred to soil.

### Plant Materials and Growth Condition

Cassava plants were grown in a greenhouse with a minimum of 14 h of light, 60% humidity, day/night temperatures of 24 and 17°C, respectively. Greenhouse grown plants were propagated via stem cuttings from the mother plants. The stem cuttings contained at least 2 buds and were planted first in Jifi pots for rooting. Within 2 months, the rooted cuttings were transferred to 15-cm round pots containing 40% Klasmann Substrate 2, 10% Perlite, 50% Ricoter lawn soil, fertilized with Scotts Osmocote. Plants were harvested for analysis after a total of 8–10 months growth. The storage root yield varied significantly between biological replicate plants. Since there was not always sufficient root material from each plant for starch extraction and all downstream analyses, we adopted a sampling strategy where equal amounts of material from each replicate plant was pooled. Although this prevents us from measuring variation between the biological replicates, this limitation is offset by the fact that we analyzed multiple independent transgenic lines and measured them over multiple generations, using independently grown batches of plants. Our strategy has the advantage that all analyses were performed on uniform sample material.

### Quantitative RT-PCR

Cassava leaves or roots were powdered in liquid N_2_. Equal amounts of powder from 3 or more plants were pooled. Samples (20 mg of leaf powder or 80 mg root powder) were vortexed with 600 μL RNA extraction buffer (150 mM Tris, pH 7.5, 2% [w/v] SDS, 50 mM EDTA). Ethanol absolute (150 μL), potassium acetate (5 M, 66 μL) and chloroform: isoamyl alcohol (24:1; 750 μL) were then sequentially added to the mix, vortexed and separated by centrifugation at 13,000 *g* for 3 min. The supernatant (600 μL) was taken and further cleaned by vortexing with 600 μL phenol:chloroform:isoamyl alcohol (25:24:1). Ethanol was added to the supernatant and the mixture was incubated at −80°C for 30 min to precipitate nucleotides. The precipitate was washed with 80% (v/v) ethanol, dissolved in 75 μL DEPC water, and RNA precipitated by addition of 25 μL 8 M LiCl in DEPC water for 16 h at −20°C. The RNA pellet was washed with 80% (v/v) ethanol, dissolved in 30 μL DEPC water and DNase I treated. Complementary DNA was synthesized using the SuperScript III kit (Invitrogen) and oligo(dT) primers. Real-time quantitative PCR was carried out using the SYBR Green Supermix (Eurogentec) with Applied Biosystem 7500/7500 Fast. PP2A was used as a reference gene. Gene-specific transcripts were normalized to PP2A and quantified by the Δ*C*t method (*C*t of gene of interest - *C*t of PP2A gene). Primer sequences (5′–3′) were as follows: *MeLSF2*, TGAGGAACCCATATGAGTACCA and GCTGCAAATTTAGAATGTAGGCC (150-bp amplicon); *MeSEX4*, ATTCAGCATCTACGTGCAGA and CTCTTCCTAGCCCAGCAGTG (145-bp amplicon); *MePP2A*, TGCAAGGCTCACACTTTCATC and CTGAGCGTAAAGCAGGGAAG (150-bp amplicon).

### Protein Extraction and Immunoblotting Detection

Powdered frozen material from pooled leaf or root samples (0.3 *g*) was homogenized in 1 mL ice-cold extraction medium (100 mM MOPS, pH 7.2, 1 mM EDTA, 10% [v/v] ethanediol, 1% [w/v] PVPP, 1 mM DTT, 1 × Complete proteinase inhibitor [Roche]) using a pre-chilled glass homogenizer. After centrifugation (10 min, 16,000 *g*, 4°C), protein in the supernatant was determined using the Bradford method (Bradford, 1976). Equal amounts of protein were separated by SDS-PAGE, elctroblotted onto polyvinylidene fluoride membranes and detected with antibodies raised against recombinant *St*GWD protein (Eurogentec, Seraing, Belgium), or against the *Arabidopsis thaliana* SEX4 (Niittylä et al., 2006).

### Iodine Staining

Ten cassava leaves (counting from the top) were harvested at the end of the day or end of the night from each plant, cleared in 80% (v/v) ethanol, rinsed in water and iodine-stained with Lugol’s solution (Sigma-Aldrich).

### Starch Quantification

The method of Smith and Zeeman (2006) was used. Briefly, powdered frozen material (leaf or root) was homogenized in 1.12 M perchloric acid. After centrifugation (3000 *g*, 10 min, 4°C) the insoluble material was washed four times in 80% (v/v) ethanol, resuspended in water and used for starch quantification. Starch in the insoluble fraction was gelatinized at 95°C for 15 min and digested to glucose at 37°C using α-amylase and amyloglucosidase (Roche). Starch content (in glucose equivalents) was determined by quantifying the released glucose with a hexokinase/glucose-6-phosphate dehydrogenase-based spectrophotometric assay.

### Starch Purification

Cassava storage roots were harvested from the wild type and from each of the independent transgenic lines. Roots from 3 or more plants per line were chopped, immediately frozen in liquid N_2_ and ground to powder using a Geno Grinder (SPEX^®^ SamplePrep, Stanmore, United Kingdom). Cassava root powder (10 mL) was homogenized in a Waring Blender in 100 mL of 50 mM Tris-HCl, pH 8.0, 0.2 mM EDTA, 0.5% (v/v) Triton X-100. Insoluble material was collected by centrifugation (15 min, 3000 *g*), resuspended in the same medium and sequentially filtered through 100 and 60 μm nylon meshes. Starch granules were purified by sedimenting through a 95% (v/v) Percoll cushion at 2,500 *g* for 15 min. The starch pellet was washed at least three times in 0.5% (w/v) SDS, and the SDS then washed away by at least three water washes. Purified starch was then dried under vacuum for 48 h. Subsequent analyses required more starch than was yielded by each plant. Therefore, equal quantities of starch from each line was pooled.

### Total Starch-Bound Phosphate Content

Purified starch granules (5 mg) were suspended in water and acid-hydrolyzed in 50 μL 2 M HCl for 2 h at 95°C. The solution was neutralized with 1 M NaOH. Fifty μL of supernatant was then treated with 15 units of Antarctic Phosphatase (2 h, 37°C). Released phosphate was determined using the malachite green assay in which is a complex formed between free phosphate, malachite green reagent and molybdate. Color formation was monitored spectrophotometrically at a wavelength of 660 nm. A standard curve correlating phosphate content and absorbance was generated using a dilution series of 0–10 nmol K_2_HPO_4_.

### ^31^P NMR Assay

Samples for ^31^P NMR analysis were prepared according to Santelia et al. (2011). Briefly, starch (50 mg) was suspend in 3 mM NaCl, 1 mM CaCl_2,_ pH 6, and digested with α-amylase and amyloglucosidase (Roche). After adjusting the sample pH to 6 and adding 5% (v/v) D_2_O, ^31^P NMR spectra were measured on an Avance III 500 MHz spectrometer equipped with a CPQCI CryoProbe with cryo-cooled ^31^P preamplifier and a *Z* axis-pulsed field gradient unit (Bruker) at 303K. ^31^P spectra of cassava starch were obtained for 5–10 k transients (3.8 s recycle delay, 1 s acquisition, 10 Hz line-broadening). All spectra were processed, analyzed and plotted with Topspin 3.2 (Bruker). ^31^P spectra were calibrated indirectly with the DSS signal as external reference with Ξ= 0.404807356 (Maurer and Kalbitzer, 1996). Wild-type Arabidopsis starch NMR was used as a reference for peak identification. Percentages of 3P and 6P peaks were obtained from integrals of baseline-corrected spectra. Errors were estimated from technical triplicates and verified for the wild type and some transgenic lines by performing analyses on replicate samples.

### Scanning Electron Microscopy (SEM)

A starch-water mix was loaded on to double-sided adhesive carbon tape mounted on SEM stub, air dried and then sputter-coated with osmium (2 nm thickness). Samples were visualized using a Hitachi SU5000 microscope at an acceleration voltage of 5.0 kV and a working distance of 6–7 mm.

### Chain Length Distribution

Purified starch (0.2 mg) was suspended in 450 μL water. Samples were heated to 100°C for 15 min and cooled to 20°C. Sodium acetate (10 mM, pH 4.8) was added together with 1 unit pullulanase and 0.04 units of isoamylase (Megazyme) to debranch the samples (37°C, 2.5 h). The reaction was stopped by heating (99°C, 10 min) and clarified by centrifugation (5 min, 16,000 *g*). The supernatant was applied to sequential 1.5 mL columns of DOWEX50 and DOWEX1 to remove the charged compounds. Neutral compounds were eluted in 4 mL water and freeze-dried. After re-dissolving in water by heating (99°C, 5 min) linear chains were separated and detected by HPAEC-PAD (ICS-5000, Dionex) using a CarboPack PA-200 column as described previously (Streb et al., 2008).

### Granule Size Distribution

Six mg of purified starch granules were suspended in 2 mL water, and sonicated for 1 min to disaggregate the granules. Granule size distribution between 3 and 30 μm was analyzed with a LS 13 320 laser diffraction particle size analyzer (Beckman Coulter). The starch granule suspension was further diluted in water to attain an obscuration level of about 10% and continuously stirred to prevent sedimentation.

### Amylose Content Assay

Purified starch in water (5 μg μL^−1^) was used for determining amylose content essentially according to Zhu et al. (2008). Samples were incubated at 98°C for 2 h with intermittent vortexing. Five μL of gelatinized sample was stained with 10% Lugol solution. The absorbance was immediately measured at 510 and 620 nm and amylose content calculated based on potato standards. A standard curve correlating amylose content and absorbance difference (620–510 nm) was generated using potato starches (Sigma) with different amylose contents (0, 10, 20, 30, 50, and 80%). The resulted equation of the standard curve was: absorbance difference (*y*) = 0.0105 amylose content (*x*) – 0.2887 (*R*^2^ for the standard curve = 0.997).

### Starch Functional Properties

Swelling power was measured using the method from Kusumayanti et al. (2015), with modifications. The starch dispersion (10 mg starch per mL water) was incubated at 55°C for 1 h with constant agitation followed by centrifugation at 371 *g* for 15 min. Swelling power was calculated according to the equation: Swelling power = weight of sediment paste/dry weight of the sample (g/g). The paste clarity was determined according to Craig et al. (1989). Pastes were produced by suspending 10 mg starch in 1 mL water and heating to 90°C for 30 min with constant agitation. After cooling to 30°C for 1 h, light transmittance of the paste was measured at 650 nm, with water as a reference. Samples were stored at 4°C and the transmittance was remeasured every 24 h. To analyze the thermal parameters of gelatinization, melting temperature (DSC-Tp) and the heat of melting (DSC-H) were determined using a Mettler-Toledo DSC with STARe evaluation software. Three mg of starch was added to 30 μL water and stirred at 20°C for 24 h. Eleven mg of the water and starch mixture was sealed into an aluminum pan for measurement. DSC was carried out in an N_2_ atmosphere at a heating rate of 5°C/min from 30 to 100°C. To evaluate paste viscosity, purified starch was analyzed using a RVA4500 Rapid Viscoanalyzer (Perten Instruments). Gelling properties were recorded by placing a 16% (w/v) starch/water slurry in the instrument and stirring for 10 s at 960 rpm and then at 160 rpm for the remaining time. The temperature profile was as follows: 50°C for 1 min, followed by a linear increase to 95°C over 3 min 42 s, hold at 95°C for 2 min 30 s, cool to 50°C over 3 min 48 s and hold at 50°C for 2 min. Viscosity was recorded continuously.

### Statistical Analysis

Two-tailed unpaired *t*-tests, used for determining significant difference between modified starches and control samples in C3 phosphate percentages and in swelling power, were conducted with the Prism software, as were Dunnett’s multiple comparison tests for amylose content and starch granule size. The least significant values were calculated at 5% probability. Relationships between starch characters and starch physico-chemical properties for GWD-expressing lines were analyzed by the means of Pearson correlation and displayed as heat map with the relevant *P*-values indicated.

### Accession Numbers

The gene NCBI and/or Phytozome accession numbers, respectively, for the following genes/promoters are: *StGWD*, XM_006357557.2 and PGSC0003DMT400019845; *MeLSF2*, XM_021769917.1 and Manes.10G005000.1; *MeSEX4* XM_021770800.1 and Manes.10G053500.1; *MePP2A*, XM_021767530.1 and Manes.09G039900.1: *S.tuberosum* B33 class I patatin promoter GQ352473.1; CaMV 35S promoters: X04879.1.

## Results

### Generation of Cassava Transgenic Plants Either Overexpressing the *StGWD* Gene, or Repressing the Endogenous *SEX4* or *LSF2* Genes

To increase the rate of starch phosphorylation we generated constructs to overexpress either the wild-type *St*GWD protein or a mutant version of it (*St*GWDm) that is insensitive to redox regulation (Mikkelsen et al., 2005) in cassava (Supplementary Figure S1A). Both genes were placed under the control of the constitutive cauliflower mosaic virus 35S promoter (CaMV 35S), and were transformed into the cassava variety TMS60444 through Agrobacterium-mediated friable embryonic callus (FEC) transformation (Taylor et al., 2004; Bull et al., 2009). Putative transformants displaying both shoot and root regeneration under antibiotic selection were analyzed for the presence of the transgenes by PCR. Thirteen independent 35S::*StGWD* lines and 35 35S::*StGWDm* lines were selected. The *St*GWD protein expression in leaves of *in vitro* grown plantlets was detected by immunoblotting using a polyclonal antibody raised against the recombinant *St*GWD protein (Eurogentec, Seraing, Belgium). The *St*GWD protein levels varied among the transgenic lines (Supplementary Figure S1B). In some cases, no transgene expression was detected and these lines served as transgenic controls. Plants were transferred to soil and grown for 8 months in the greenhouse. The transgenic lines grew well but were ∼15% shorter than untransformed wild-type plants. The fresh weight of the storage roots in the transgenic lines was also ∼40% lower than in the untransformed plants, although the roots were similar in appearance. However, plant growth and storage root yield were comparable within the set of transgenic plants, suggesting a general influence of the cassava transformation and regeneration process on subsequent growth, compared with untransformed wild-type plants. It is also important to note that our greenhouse cultivation conditions cannot be used to obtain a valid measure of agronomic performance for cassava.

**FIGURE 1:**
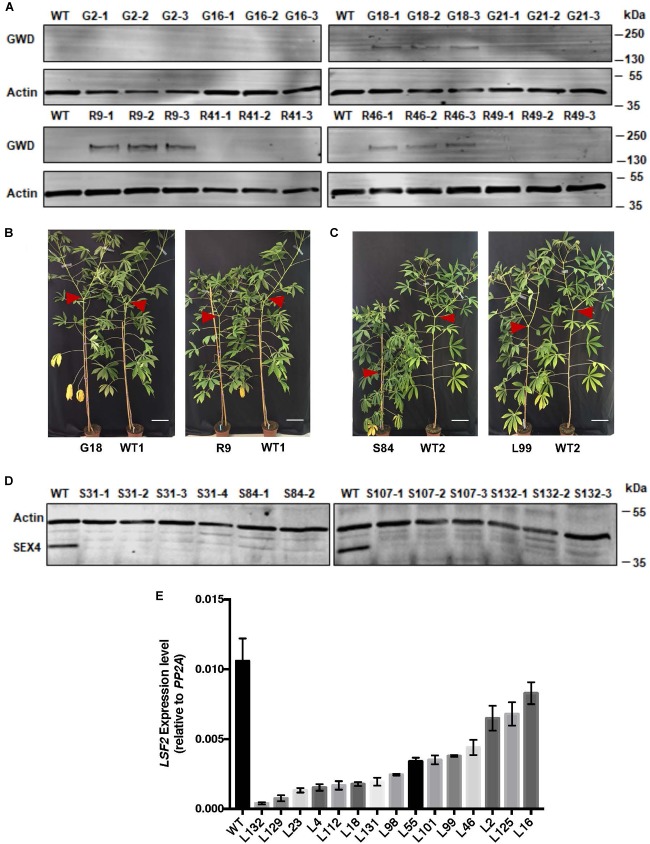
Analysis of gene expression in transgenic cassava lines. **(A)** Immunoblot detection of GWD proteins (G, wild-type *St*GWD protein; R, redox insensitive version) in soluble protein extracts from cassava storage root (5 μg protein loaded). Wild-type (WT) control and 3 technical replicates of pooled material (from 3 or more individuals of each transgenic line) were analyzed. Actin (loading control) was detected on the same membrane. **(B)** Growth comparison of representative G- and R-lines. **(C)** Growth comparison of representative *LSF2* RNAi lines (L-lines) and *SEX4* RNAi lines (S-lines). Red arrows indicate shoot branching. Bar = 15 cm. **(D)** Immunoblot detection of the *Me*SEX4 protein in soluble protein extracts (5 μg protein loaded). Wild-type control and 2–4 technical replicates of pooled material (from 3 or more individuals of each transgenic line) were analyzed. Actin was used as a loading control. **(E)** Expression of the *LSF2* gene in storage roots relative to the *PP2A* reference gene determined by RT-PCR. Mean values (±SD) of three replicate analyses of pooled material (from 3 or more individuals of each transgenic line). These experiments were repeated three times with similar results with independently grown populations of plants.

Of the 35S::*StGWD* lines (hereafter referred to as ‘G’ lines), G18, G2 (high expression), G16 (lower expression), and G21 (transgenic control) were selected for further analysis. From the 35S::*StGWDm* lines (hereafter referred to as ‘R’ lines), R9, R41, R46 (high expression), and R49 (transgenic control) were selected. In storage roots, the expression level was in some cases similar to that in leaves, but not in all of them. Of the G-lines, only G18 had detectable levels of *St*GWD protein while among the R-lines, both R9 and R46 had detectable levels of protein in storage roots (Figure 1A).

To decrease the rate of starch dephosphorylation, we generated RNAi hairpin constructs against either *MeSEX4* or *MeLSF2* gene and expressed them under the control of the *S. tuberosum* patatin B33 promoter (Supplementary Figure S2), which is known to drive expression in roots of cassava and Arabidopsis (Ihemere et al., 2006; Naumkina et al., 2007). The constructs were transformed into TMS60444, again via Agrobacterium-mediated transformation. Antibiotic selection and screening for the presence of the transgene by PCR on genomic DNA resulted in 20 independent *MeSEX4* RNAi lines and 30 independent *MeLSF2* RNAi lines. The *MeSEX4* lines (hereafter referred to as ‘S’ lines) displayed severe growth phenotypes (Figure 1B), being dwarfed and slow-growing. Only four lines (S31, S84, S107, and S132) grew sufficiently well to be stably propagated in soil (Figure 1C) and analyzed further. In contrast, the *MeLSF2* lines (hereafter referred to as ‘L’ lines) grew well, like the GWD-expressing plants (Figure 1C). The expression of the targeted gene was analyzed for each line. Immunoblots using an antibody raised against the *A. thaliana* SEX4 protein revealed a protein of the predicted molecular weight of *Me*SEX4 in extracts of storage roots of wild-type cassava. This protein was efficiently silenced in the storage roots of all four S-lines (Figure 1D). Since an antibody recognizing the *Me*LSF2 protein was not available, RT-PCR was used to assess *MeLSF2* gene expression. This revealed efficient transcriptional repression in the storage roots of most of the L-lines (Figure 1E). Gene expression in leaves of selected S- and L-lines was also checked by RT-PCR. This revealed wild-type levels of the *MeSEX4* transcript in the S-lines (Supplementary Figure S3A), and either wild-type levels or a small decrease in *MeLSF2* transcripts for the L-lines (Supplementary Figure S3B).

### Starch Phosphate Content and Distribution Are Significantly Altered in Storage Roots of Transgenic Cassava Lines

**FIGURE 2:**
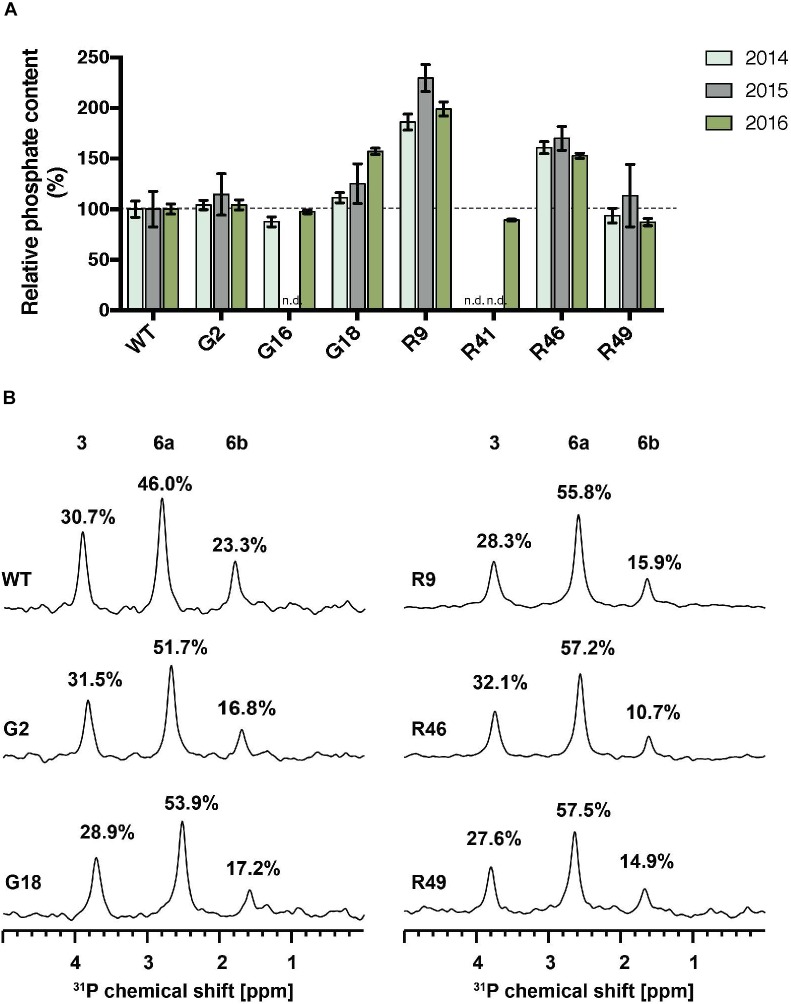
Total phosphate content and C3:C6 phosphate distribution on starch of GWD overexpression lines. **(A)** Starch was purified from storage roots of the indicated lines in three consecutive harvests. Total starch bound phosphate was measured with the malachite green assay. The phosphate content relative to the wild type (WT) is shown, ±SD. The mean phosphate content of WT was 0.62, 0.31, and 0.38 nmol P/μmol Glc in 2014, 2015, and 2016, respectively. For each line and for each harvest, three technical replicates were measured using pooled starch samples from 3 or more plants. n.d., not determined. **(B)** ^31^P-NMR one-dimensional spectra of hydrolyzed starch was performed to detect C3-bound (3) and C6-bound (6a and 6b) phosphates. Peak areas are proportional to the relative amount of phosphate and are given as a percentage above each peak.

The total phosphate content of starch purified from storage roots was determined using the malachite green assay. Significant increases in starch-bound phosphate were observed in the G- and R-lines, where *St*GWD protein was detected in the storage root (Figures 1A, 2A). The highest level was observed in line R9, which had around twice as much starch-bound phosphate as the wild type in all three harvested generations (Figure 2A). R46 had increases above 50%, and G18 had a significant increase in 2016 but not in the preceding 2 years. In the low-expressing lines G2, G16, R41, and R49, there was no significant increase in phosphate content (Figure 2A). To investigate the distribution of starch-bound phosphate between the C3 and the C6 positions, ^31^P NMR was performed. All selected G- and R-lines showed similar ratios of C3:C6 phosphate as in the wild type (Figure 2B). This is interesting since GWD specifically phosphorylates at the C6 position, while PWD phosphorylates the C3 position.

**FIGURE 3:**
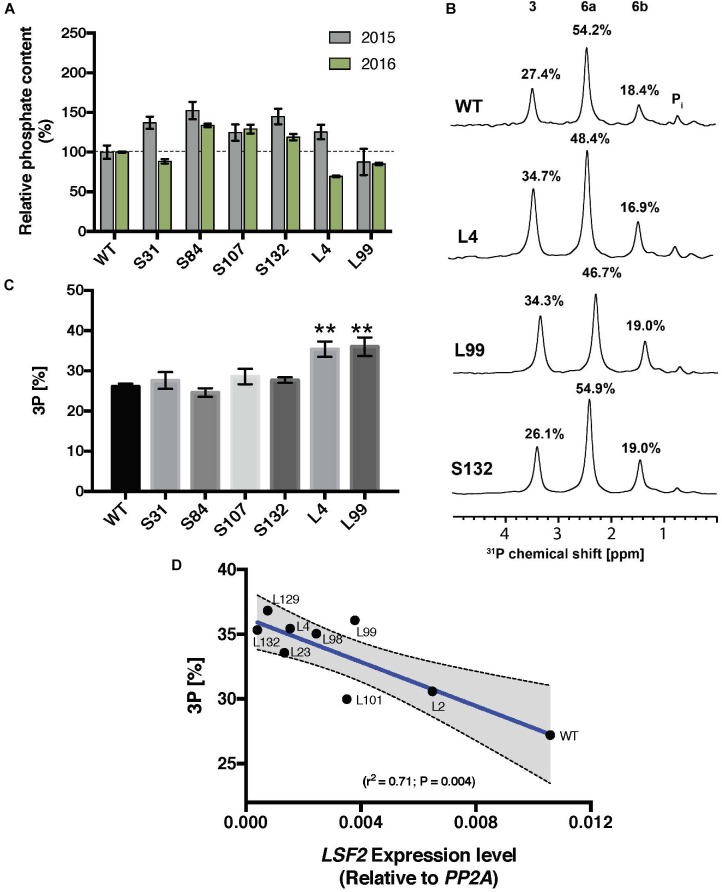
Total phosphate content and C3:C6 phosphate distribution on starch of *MeSEX4* RNAi and *MeLSF2* RNAi cassava lines. **(A)** Starch was purified from storage root of representative S and L lines in 2015 and 2016 and dried. Total starch-bound phosphate was measured and is expressed as a percentage relative to the wild-type value for each year. Values are means ± SD of three technical replicates from pooled starch samples (from 3 or more plants of each line and for each harvest). **(B)** Example ^31^P-NMR one-dimensional spectra of hydrolyzed starch, performed to detect C3-bound (3) and C6-bound (6a and 6b) phosphates. Peak areas are proportional to the relative amount of phosphate and are given as a percentage above each peak. Pooled samples from 3 or more plants of each line were used. **(C)** Percentage of C3-bound phosphate on starch of selected lines, measured through ^31^P-NMR, as above. Values are the means ± SD of three replicate samples. Student’s *t*-tests were performed; ^∗∗^*P* < 0.01. **(D)** Correlation between the percentage C3-bound phosphate and *LSF2* gene expression in indicated L-lines. Blue line indicates the linear correlation, with 95% confidence intervals. Error bands (dashed lines) and error area (gray) are shown. For **(B–D)**, data shown are for the 2015 harvest. Similar results were obtained for selected lines in the 2016 harvest.

For the S-lines, the total starch-bound phosphate was slightly increased in three of the four cases over two generations, whereas in the L-lines, the level was similar to the wild type (Figure 3A). In the S-lines, no alteration in the ratio of C3:C6 phosphate was detected whereas in the L-lines, L4 and L99, there was a significant increase in the proportion of C3-bound phosphate (from 27% in the wild-type to 35% in these *MeLSF2* RNAi lines; Figures 3B,C). Analysis of additional L-lines (L2, L23, L98, L101, L129, and L132) further confirmed the relative increase in C3-bound phosphate and established that this parameter was inversely correlated with the expression level of the *MeLSF2* gene (*r*^2^ = 0.71, P = 0.004; Figure 3D).

### Starch Structure and Granule Morphology Are Unaffected by Altered Phosphate

**FIGURE 4:**
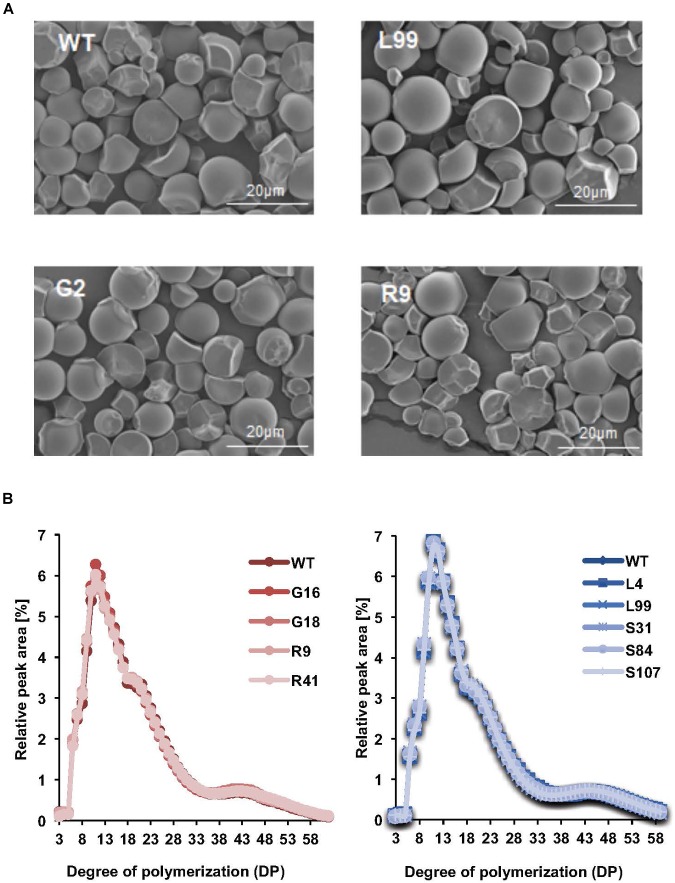
Starch structure in transgenic cassava roots. **(A)** Scanning electron micrographs of purified and dried root starch granules from pooled starch samples from the 2016 harvest. Line names and scale bars are indicated. **(B)** Comparison of chain length distributions of starch in different transgenic lines. Left is the CLDs of G- and R-line starches from samples in 2016. Right is the CLDs of S and L line starches from samples in 2015. Values are means of four technical replicates performed on starch samples pooled from 3 or more plants.

The starch granules from the storage roots of our transgenic cassava, viewed by scanning electron microscopy, were irregularly shaped, with ovoid, polygonal and round granules observed. The starch granule surfaces were predominantly smooth with some concave pits (Figure 4A). There were no obvious differences in the appearance of the starch granules among the transgenic and the wild-type samples. The micrographs showed that the granule size ranged from 3 to 30 μm. Further quantification of the particle size distributions within this range by laser diffraction indicated that the modal particle size was 12.4 μm (Figure 4A and Table 1). Similar granule sizes were detected for the transgenic lines (modal values ranging from 10.3 to 13.6 μm; Table 1).

**Table 1:** Starch compositional characteristics in transgenic cassava roots.

Construct	Line	Starch content (mean mg.g^−1^ FW)	Amylose content (mean %)	Starch granule size (mode, μm)
*StGWD*	**WT**	269.5 (±9.9)	19.0 (±2.6)	12.4 (±1.4)
&*StGWDm*	**G2**	199.5 (±4.9)	16.9 (±0.4)	13.6 (±1.4)
OE	**G16**	239.5 (±12.4)	18.1 (±0.3)	13.6 (±1.4)
	**G18**	210.9 (±10.3)	18.8 (±0.9)	13.6 (±1.4)
	**R9**	215.4 (±5.3)	18.2 (±0.8)	12.4 (±1.4)
	**R41**	247.1 (±8.2)	19.0 (±0.4)	12.4 (±1.4)
*MeSEX4*	**WT**	277.8 (±8.7)	20.3 (±0.5)	12.4 (±1.4)
&*MeLSF2*	**S31**	241.2 (±10.2)	17.9 (±0.7)	10.3 (±1.4)
RNAi	**S84**	193.7 (±11.1)	18.9 (±0.7)	11.3 (±1.4)
	**S107**	211.4 (±7.9)	18.8 (±1.0)	11.3 (±1.4)
	**L4**	327.2 (±13.2)	22.1 (±0.7)	13.6 (±1.3)
	**L99**	166.5 (±5.6)	20.2 (±1.0)	10.3 (±1.3)

*Mean or mode values (±SD of three technical replicates) are given.*

Amylose content – an important factor influencing starch properties – was determined in the storage root starch of our transgenic lines. Amylose content ranged from 16.9 to 22.1%, but there were no statistically significant difference amongst the transgenic lines with the exception of line S31, the amylose content of which was slightly lower than that of wild-type plants (Table 1). We also analyzed the chain length distribution (CLD) of the starches to provide insight into branched amylopectin architecture. After enzymatic debranching, the resultant linear glucan chains were analyzed by high performance anion exchange chromatography with pulsed amperometric detection (HPAEC-PAD). The distribution of chains ranging in length from DP (degree of polymerization) 3 to 60 revealed a modal value of DP 13 (Figure 4B). No difference in distribution was observed among the transgenic lines compared to the wild type (Figure 4B). Thus, the starch characteristics we tested here (granule size, granule surface morphology, amylose content and chain length distribution) were generally not influenced by the alteration of phosphate content.

### Cassava Starch Physico-Chemical Properties and Functionality Are Influenced by Phosphate Content but Not Phosphate Position

**FIGURE 5:**
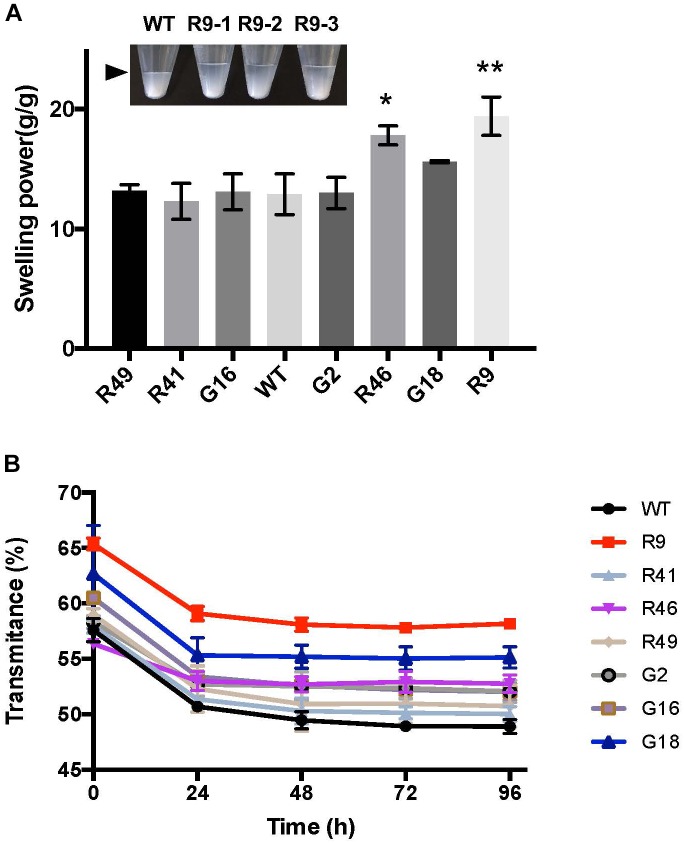
Swelling power and starch paste clarity of GWD over-expression lines. **(A)** Swelling power of starch from GWD over-expression lines. The pooled starch from lines R46 and R9 had significantly increased swelling power (*t*-test; ^∗^*P* < 0.05, ^∗∗^*P* < 0.01). Inset: picture of R9 line with three replicates showing the level of swollen starch (arrowhead). Starches were purified from samples harvested in 2016. **(B)** Starch paste clarity was determined by light transmittance at 650 nm of gelatinized starch stored at 4°C for the given 24 h periods. Starches were purified from samples harvested in 2016. All values are the means ± SD of three technical replicates performed on pooled starch from 3 or more plants.

Alteration of the number and/or distribution of negatively charged phosphate groups might influence starch functional properties. To investigate whether the variation in the amount of starch-bound phosphate in our cassava lines changed starch swelling power, we heated samples of starch (from the G- and R-lines) that contained different amounts of phosphate in water at 55°C. The weight of wet sediment formed during heating was determined and the swelling power calculated based on the increased weight. The line with the highest phosphate, R9, had a significantly higher swelling power (19.4 g/g) compared to the wild type (12.9 g/g; Figure 5A). Starches from lines R46 and G18, which also had high starch-bound phosphate, similarly showed increased swelling powers (17.8 and 15.6 g/g, respectively). Starches from the other transgenic lines, where there was no increase in starch-bound phosphate (G2, G16, R41, and R49) had swelling power similar to wild-type starch (Figure 5A).

Next we analyzed paste clarity – another important starch property. After gelatinization and initial measurement of clarity (assessed with light transmittance), the starch was stored at 4°C and the clarity re-measured over the subsequent 4 days. Light transmittance gradually decreased due to retrogradation, reaching a stable level after 48 h. While this pattern was observed in all our cassava lines (Figure 5B), the pastes of the high-phosphate starches (R9, R46, and G18) displayed an improved initial and/or final light transmittance (Figure 5B). This suggests that the charged phosphate groups help to keep starch molecules hydrated and reduce the extent to which they re-crystallize.

Differential scanning calorimetry (DSC) was used to determine starch thermal properties during gelatinization. No significant correlation was observed between starch phosphate content and gelatinization temperature or enthalpy (Supplementary Table S1). Among all selected lines, DSC peak temperatures ranged from 59.8 to 62.8°C and the gelatinization enthalpies ranged from 15.1 to 19.2 Jg^−1^ (Supplementary Table S1). There was a negative correlation between DSC gelatinization temperature and enthalpy amongst the samples.

**FIGURE 6:**
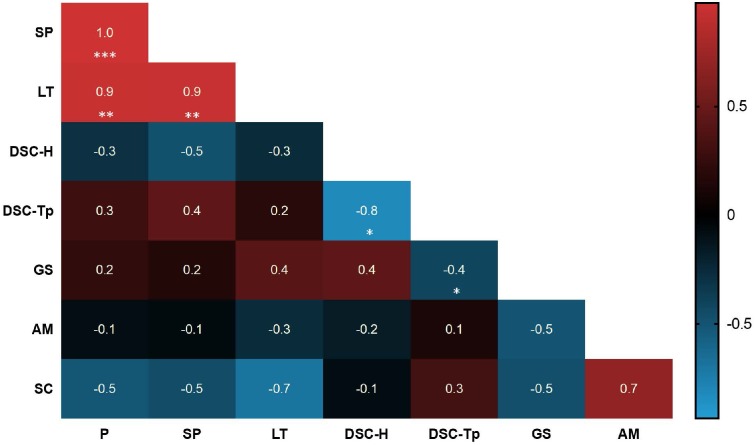
Heatmap displaying the Pearson correlation between starch characters and starch physicochemical properties. Each correlation value is indicated and the relevant *P*-value is shown as significance. The asterisks indicate significance (^∗^*P* < 0.05, ^∗∗^*P* < 0.01, ^∗∗∗^*P* < 0.001). Red colors show positive correlations and blue colors show negative correlations. P, phosphate content; SP, swelling power; LT, light transmittance (after 96 h storage); DSC-H, DSC gelatinization enthalpy; DSC-Tp, DSC gelatinization peak temperature; GS, granule size; AM, amylose content; SC, starch content.

An overview of the significantly correlated physico-chemical properties (swelling power, paste clarity and DSC) and compositional characteristics (phosphate content, granule size and amylose content) of starch strongly suggest that the phosphate content of cassava starch influences swelling power (*r* = 1.0, *P* < 0.001) and paste clarity (*r* = 0.9, *P* < 0.01), but not gelatinization temperature, granule size or amylose content (Figure 6). We also performed rapid viscometric analyses (RVA) of selected G-and R-line starches. While there was some variation in the viscosity profiles, this did not correlate with phosphate content (Supplementary Figure S4).

**FIGURE 7:**
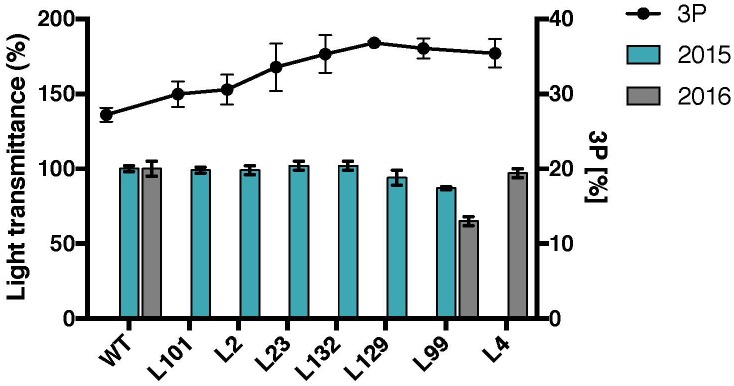
Starch paste clarity and C3-bound phosphate percentages of selected *MeLSF2* RNAi cassava lines. C3-bound phosphate (3P) percentage (right *Y*-axis) was measured through ^31^P-NMR (same values as presented in Figure 3D). The mean value (±SD) of three replicate analyses of pooled starch were included. Light transmittance of gelatinized starch (measured after 96 h – as in Figure 5) from root samples harvested in 2015 and 2016. Values are expressed relative to the wild-type (WT) transmittance (left *Y*-axis) and are the means ± SD of three replicate analyses of starch pooled from 3 or more plants of each line.

To investigate whether the variation of the ratio of C3-bound to C6-bound phosphate results in any changes in starch properties, the *MeLSF2* RNAi lines with different levels of C3-bound phosphate were analyzed. No major changes in light transmittance were detected among the lines, except for a slight decrease in L99 (Figure 7). The other starch physico-chemical properties (swelling power and DSC-based gelatinization) of L-lines were also tested, but no significant correlations were observed (data not shown). Thus, unlike phosphate amount, there is no significant change in cassava starch properties resulting from the distribution of the phosphate between C6 and C3 positions.

## Discussion

This work demonstrates that, using transgenic methods, it is possible to increase the phosphate content of cassava storage root starch. This leads to important changes in starch properties that can be valuable for industries that use starch as a raw material. In addition, this work shows that targeting genes discovered and studied in Arabidopsis leaves it is possible to change the relative abundance of phosphate at the C3 and C6 positions of the glucosyl residues of cassava starch amylopectin. This, together with another very recent study of the role of LSF2 in potato tubers (Samodien et al., 2018), demonstrates a conserved function between species and plant tissues.

### Control of Starch Phosphorylation in the Storage Root of Cassava

The particularly high levels of covalently bound phosphate in potato starch (reportedly 4–10 times higher than cassava root starch) led us to overexpress the *St*GWD gene based on the hypothesis that it may be fully or partly responsible for this potato starch trait. Further, we also over-expressed a mutant version of the *St*GWD protein to circumvent potential redox-regulation in the storage root amyloplasts. In several cassava lines, expression of either proteoform successfully increased the amount of starch-bound phosphate. This is consistent with the findings in previous studies on other crops, such as barley (Carciofi et al., 2011) and rice (Chen et al., 2017), where the starch phosphate levels were increased by overexpressing *St*GWD – in those cases in the seed starchy endosperm.

In our cassava transformants, starch-bound phosphate in the storage roots was elevated twofold compared to wild-type cassava (Figure 2A). This is still not as high as the amounts seen in potato tuber starch, suggesting that factors other than the *St*GWD itself contribute to the high levels. Such other factors could include the concentration of ATP, which may be higher in potato amyloplasts than in cassava root amyloplasts. Alternatively, differences in the structure of amylopectin between potato and cassava may affect the degree to which *St*GWD acts. It was shown previously that GWD has much higher activity on branched substrates with longer rather than shorter chains (Mikkelsen et al., 2004). Consistent with this, cassava amylopectin has a shorter average chain length than potato (Waterschoot et al., 2014a). It is also possible that the other enzymes known to phosphorylate or dephosphorylate starch play a role in determining the overall phosphate level. Our data suggest that this latter point is at least part of the explanation. For example, in our *St*GWD-overexpressing lines, the ratio of phosphate bound to the C6 and C3 position of the glucosyl residues of amylopectin remained the same as in the wild type (Figure 2B). This means that an increased phosphate at the C3 position was observed when overexpressing the *St*GWD protein. It is known that C6- and C3-phosphorylation is selectively catalyzed by GWD and PWD, respectively (Ritte et al., 2006), and that PWD is strictly dependent on the pre-phosphorylation by GWD (Baunsgaard et al., 2005; Kötting et al., 2005). Thus, it appears that the activity of endogenous *Me*PWD is increased in *St*GWD overexpression lines leading to the higher phosphorylation at the C3 position.

The phosphoglucan phosphatases SEX4 and LSF2 have been shown to influence both the amount and distribution of starch-bound phosphate in Arabidopsis. Our work shows that some of these findings can be transposed to cassava. Repression of *MeLSF2* expression increased the ratio of C3-bound to C6-bound phosphate. Multiple, stably propagated lines with different degrees of *MeLSF2* repression were obtained, in which a correlated increase in the proportion of C3-bound phosphate was evident (Figure 3D). These results are consistent with the findings in Arabidopsis, where LSF2 was shown specifically to remove the C3-bound phosphate. In that system, the increase in C3:C6 ratio in the *lsf2* mutant was greater than was observed here, and it was accompanied by an increase in the total starch bound phosphate. In potato tubers, silencing LSF2 yielded similar findings, with both an increase in C3:C6 ratio and a slight increase in total starch-bound phosphate (Samodien et al., 2018). It seem likely that the magnitude of the differences observed in each case can be explained by whether LSF2 expression is repressed (i.e., in cassava and potato) or completely abolished (i.e., in Arabidopsis).

Our results with *MeSEX4* RNAi lines were mixed and not easily interpretable. In contrast to all of the other transgenic lines, these plants grew very poorly during regeneration *in vitro* and very few survived the transfer to soil. The four successfully transferred lines continued to grow poorly on soil. The growth of Arabidopsis *sex4* mutants was also slower than the wild type (Zeeman et al., 1998), and was accompanied by a strong starch-excess phenotype in the leaves. In that case, the incomplete mobilization of stored reserves is thought to limit vegetative growth (Stitt and Zeeman, 2012). In our cassava lines, *MeSEX4* expression was decreased only in the storage roots and not in the leaves (Supplementary Figure S3A), and no starch-excess phenotype was observed in leaves either by qualitative iodine staining or quantitative measurements (data not shown). Nevertheless, the phosphate levels in the storage root starch were slightly increased, although the strength of this effect was somewhat dependent on the year of harvest (Figure 3A). Repression of SEX4 in potato also led to an increase in the phosphate content of the starch (Samodien et al., 2018). This is interesting because Arabidopsis *sex4* mutants do not have increased amount of bound phosphate per gram of leaf starch. Nevertheless, total glucan bound phosphate is still higher in the *Arabidopsis* mutant, since the starch content is increased and because large amounts of phosphooligosaccharides accumulate during starch degradation (Kötting et al., 2009; Santelia et al., 2011). We propose that differences in starch metabolism between leaves and storage organs could explain the observed differences in the types of phosphoglucans that accumulate in these tissues when amounts of SEX4 proteins are reduced (Figure 3A). Presently, the cause of the poor growth phenotype resulting from *MeSEX4* repression remains unclear. More work will be required to establish if it is a direct effect of the repression of the *MeSEX4* gene, and if so, to identify the affected tissue and reason for growth retardation.

### Impact of Starch Phosphorylation on Cassava Starch Properties

Phosphate esters are hydrophilic and can enhance starch hydration. Indeed, it is thought that the major biological function of starch phosphorylation is the solubilization of the semi-crystalline structures of starch to assist and control its degradation. Thus, it is not surprising that the presence of phosphate groups lead to changes in the valuable physico-chemical properties of starch (Jobling, 2004). Our study provides a clear picture of this influence since the elevation of starch-bound phosphate in storage root starch (e.g., line R9) occurred without affecting other features of starch. This is important since amylopectin structure, amylose content, and starch granule morphology are all well known to significantly influence its functional properties (Copeland et al., 2009). Furthermore, it is quite common for genetic perturbations to affect multiple starch traits at once. For example, in rice and barley, ten-fold increases in starch-bound phosphate were achieved by overexpression of the potato GWD in the developing endosperm. This altered starch physico-chemical properties such as gelatinisation enthalpy and also changed features such as starch granule morphology and amylose content (Carciofi et al., 2011; Chen et al., 2017). Similarly, repression of *SEX4* or *LSF2* in potato altered amylopectin chain-length distribution and starch granule size in addition to altering phosphate content (Samodien et al., 2018).

In our study, the two-fold increase in starch-bound phosphate was more modest than was reported in GWD-overexpressing cereal grains (partly because cassava starch naturally has more bound phosphate than that of rice or barley; Blennow et al., 1998; Carciofi et al., 2011). Our analysis of amylopectin structure, amylose content, and starch granule morphology revealed that they were essentially unchanged, allowing us to attribute the altered physico-chemical properties directly to the phosphate content. The swelling power of starch and the clarity of the starch pastes, two desirable traits in both the food and non-food industries (Singh et al., 2003; Waterschoot et al., 2014b; Alcázar-Alay and Meireles, 2015), were both strongly and positively correlated with starch phosphate content (Figures 5, 6), while other, gelatinisation-related properties were unaffected.

Our study also allows us to propose that it is the amount of starch-bound phosphate, rather than its distribution between C3 and C6 positions of the glucosyl residues, that is most important for its functional properties (Figure 7). On one hand, this is surprising since the C3-bound phosphate is proposed to have a greater disruptive impact on the semi-crystalline packing of native starch (Hansen et al., 2009). On the other hand, it is perhaps unsurprising that a trait like paste clarity – determined by physical processes that occur after starch gelatinization (where the natural semi-crystalline starch structure is lost) is insensitive to the location of the phosphate.

### Potential for Improved Cassava Starch Through Transgenic and Non-transgenic Means

Our data demonstrate that it is feasible to alter the phosphate content of cassava root starch and thereby its functional properties by over expressing redox insensitive *St*GWD and to change the phosphate distribution of cassava root starch by repressing *Me*LSF2. This work was performed on the cultivar TMS60444, which is amenable to genetic transformation, but not widely grown in agriculture. Future approaches using similar RNAi-based methodologies in other, farmer-preferred cultivars could help to create higher value cassava crops. It is also plausible that alternative CRISPR/Cas9-based strategies could be used, either for mutation (e.g., of the *LSF2* gene) or for targeted genome editing (e.g., to alter the expression and/or regulation of the *MeGWD* and *MePWD* genes), to create similarly improved, transgene-free cassava lines (Bull et al., 2018). Furthermore, while we have studied each gene target one at a time, a multi-target approach to simultaneously increase phosphorylation and decreased dephosphorylation may be even more successful in elevating starch bound phosphate. This may allow levels seen in potato or even higher to be achieved, gaining even greater value and broader applications for cassava in the starch industry.

## Author Contributions

WW, CH, and SZ conceived the project. WW performed most of the experiments, except for the generation of *StGWD*, *MeSEX4*, and *MeLSF2* cassava transgenic lines, which was done by CH, the ^31^P NMR analysis, which was done by FD, and the RVA analysis, which was conducted by JL and JK. WW and SZ wrote the manuscript with input from all co-authors.

## Conflict of Interest Statement

The authors declare that the research was conducted in the absence of any commercial or financial relationships that could be construed as a potential conflict of interest.

## Footnotes

1 www.phytozome.net
