# Supplementary material for: Modification of Cassava Root Starch Phosphorylation Enhances Starch Functional Properties
Source: Front Plant Sci. 2018 Oct 30;9:1562. doi: 10.3389/fpls.2018.01562 (PMC6218586; doi:10.3389/fpls.2018.01562)
Supplement: Supplementary file 1 [file Data_Sheet_1.pdf]

Supplementary Material

Modification of Cassava Root Starch Phosphorylation Enhances Starch Functional Properties.

Wuyan Wang, Carmen E. Hostettler, Fred F. Damberger, Jens Kossmann, James R. Lloyd, Samuel C. Zeeman*

*** Correspondence:** Prof. Samuel C. Zeeman: szeeman@ethz.ch

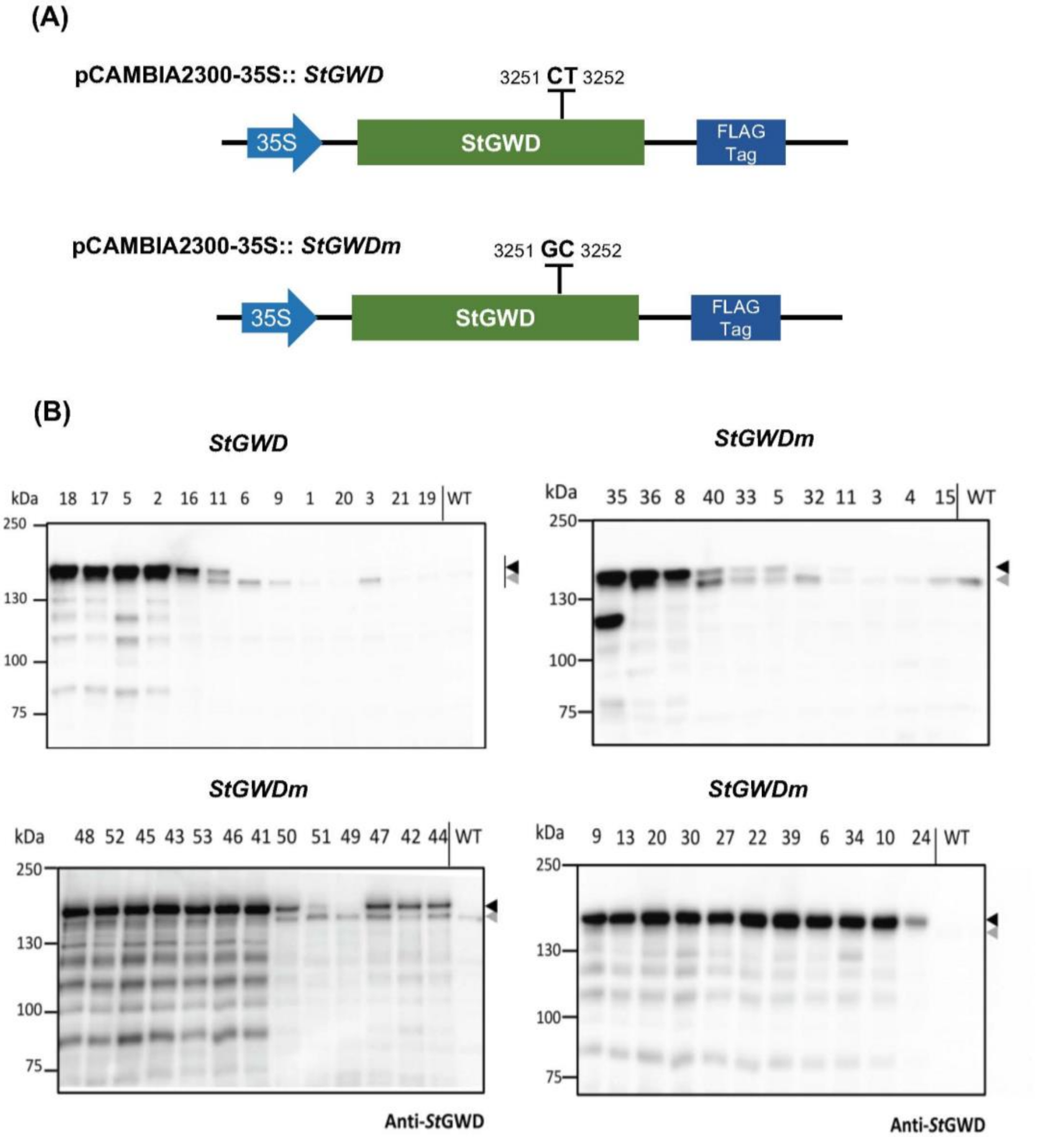

Supplementary Figure 1. Generation of transgenic cassava expressing *Solanum tuberosum* GWD. (A) Two pCAMBIA2300 plasmid constructs were made, harboring either the wild-type (pCAMBIA2300::*StGWD*) or redox-insensitive (pCAMBIA2300::*StGWDm*) coding sequence of *S. tuberosum* GWD, under the control of CaMV 35S promoter. Nucleotide modifications at location 3251 and 3252 of *StGWDm* are indicated. (B) Thirty µg of total leaf protein from individual lines were subjected to immunoblot analysis using anti-*St*GWD antibody. The black arrow indicates the *St*GWD protein. The grey arrow represents endogenous *Me*GWD protein.

(A) pCAMBIA1301-patatin:: *MeSEX4* hairpin
(or MeLSF2)

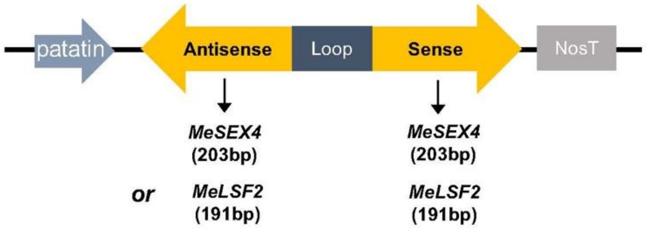

(B) cassava4.1_009735m 203bp CDS for *MeSEX4* hairpin construct

GGAGGACAAGGGAAAGTCTGAGATATACAGTCATAATATGACAGAAGCTATGGGTGC
TGTTTTGACCTATAGGCATGAACTAGGAATGAACTTCAACTTCATTTGTCCAGATTTGA
TTGTAGGATCATGCCTACAGACTCCTGAAGATGTTGACAAGCTTCGAGAAATAGGAGT
GAAAACAATATTCTGCTTGCAACAGGACC

(C) cassava4.1_013314m 191bp CDS for *MeLSF2* hairpin construct

TCTCATACCCGAATGAAACTGAAGTGTTCTTCACAAGAAATAAATCTACATGGAAATT
CTTGCTTTCGAAGGATTGCTTCAAAATGGGTAGGATCAATTGTAAGCTATCAGATAGT
GGAATCGAGAAAAAACCCACTGGAAAAGATGTGTCATTGAGCTCAACGAACAGGATG
GAAGAGTACAATACAGCC

Supplementary Figure 2. Generation of *MeSEX4* RNAi and *MeLSF2* RNAi transgenic cassava.

(A) Construct design for the production of *MeSEX4* and *MeLSF2* RNAi cassava lines. The hairpin construct contains an antisense and a sense region (203bp of coding sequence of the *MeSEX4* gene or 191bp of the coding sequence of *MeLSF2*) with a loop in the middle. The hairpin sequence was sub-cloned to vector pCAMBIA1301 and expression was driven by the *S. tuberosum* class 1 patatin B33 promoter. (B) The sequence targeting *MeSEX4* gene. (C) The sequence targeting *MeLSF2* gene.

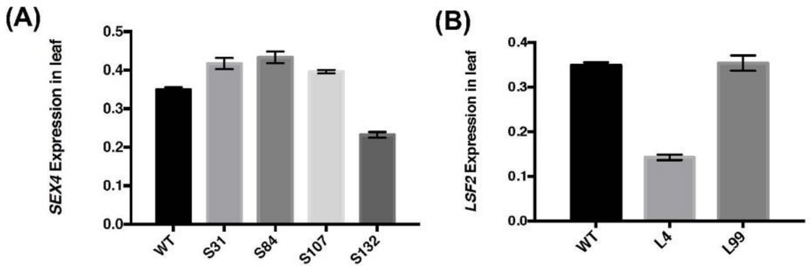

Supplementary Figure 3. *MeSEX4* and *MeLSF2* expression in the leaves of transgenic cassava lines.

(A) RT-PCR was performed using RNA extracted from leaves of *in-vitro* grown plantlets of the wild type (WT) and selected *MeSEX4* RNAi-lines. Mean *SEX4* expression levels (± SE, n=3) are given relative to the housekeeping gene *PP2A*. (B) *LSF2* expression levels in the WT and selected *MeLSF2* RNAi-lines are given relative to *PP2A*, as described in (A).

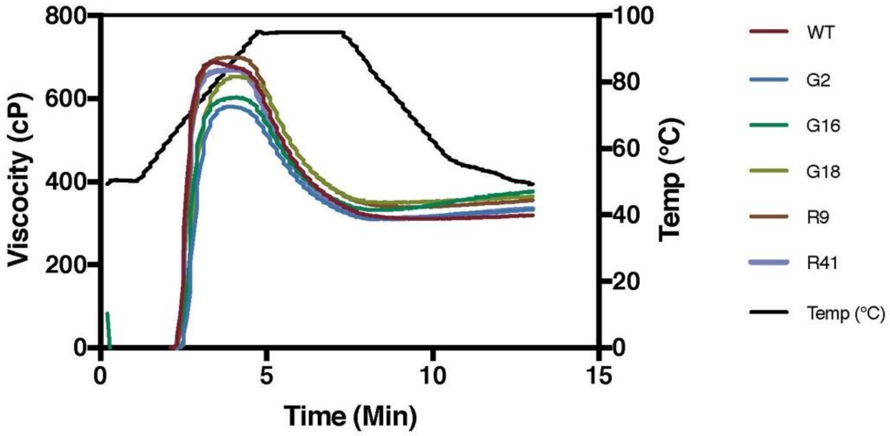

Supplementary Figure 4. Rapid viscometric analysis of starch extracted from storage roots of wild-type cassava and transgenic lines over-expressing GWD.

Viscosity profiles of starch/water slurries during heating and cooling in a rapid viscoanalyser. Each profile is the mean of three replicate measurements made for starch pooled from three biological replicates for each line.

Supplementary Table 1. Differential scanning calorimetry analysis of starch slurries from wild-type cassava and transgenic lines expressing *St*GWD.

For the DSC assay, starch from three biological replicate plants was pooled, and three replicates samples were taken. Peak temperature and enthalpy were determined using STARe software.

DSC Samples	WT	R9	R41	R46	R49	G2	G16	G18
**Peak Temp. (°C)**	61.7 ± 0.07	62.8 ± 0.13	61.2 ± 0.21	59.8 ± 0.14	61.4 ± 0.07	60.6 ± 0.17	61.0 ± 0.17	59.8 ± 0.05
**Enthalpy (Jg-1)**	17.4 ± 4.4	15.1 ± 1.3	18.6 ± 1.9	17.6 ± 2.7	15.4 ± 0.4	18.7 ± 1.9	18.2 ± 3.5	19.2 ± 1.6
